# Design and Optimization of Planar Spiral Coils for Powering Implantable Neural Recording Microsystem

**DOI:** 10.3390/mi14061221

**Published:** 2023-06-09

**Authors:** Jie Luo, Ruifeng Xue, Jiahao Cheong, Xuan Zhang, Lei Yao

**Affiliations:** 1 School of Microelectronics, Shanghai University, Shanghai 201800, China; shu_luojie@shu.edu.cn; 2 Shanghai Industrial Technology Research Institute, Shanghai 201899, China; rfxue1977@hotmail.com (R.X.); jiahao.cheong@sitrigroup.com (J.C.); zhangxuan@mtrix.cn (X.Z.)

**Keywords:** wireless power transfer, inductive coupling, planar spiral coil, resonant load transformation, implantable, neural recording microsystem

## Abstract

This paper presents a design and optimization method utilizing inductive coupling coils for wireless power transfer in implantable neural recording microsystems, aiming at maximizing power transfer efficiency, which is essential for reducing externally transmitted power and ensuring biological tissue safety. The modeling of inductive coupling is simplified by combining semi-empirical formulations with theoretical models. By introducing the optimal resonant load transformation, the coil optimization is decoupled from an actual load impedance. The complete design optimization process of the coil parameters is given, which takes the maximum theoretical power transfer efficiency as the objective function. When the actual load changes, only the load transformation network needs to be updated instead of rerunning the entire optimization process. Planar spiral coils are designed to power neural recording implants given the challenges of limited implantable space, stringent low-profile restrictions, high-power transmission requirements and biocompatibility. The modeling calculation, electromagnetic simulation and measurement results are compared. The operating frequency of the designed inductive coupling is 13.56 MHz, the outer diameter of the implanted coil is 10 mm and the working distance between the external coil and the implanted coil is 10 mm. The measured power transfer efficiency is 70%, which is close to the maximum theoretical transfer efficiency of 71.9%, confirming the effectiveness of this method.

## 1. Introduction

In recent years, implantable medical devices have played an increasingly important role in the monitoring and treatment of various diseases [1]. How to power such implantable medical devices safely and reliably is a popular current research topic. Traditional percutaneous wires require penetration of the skin, and battery power supplies are limited by their capacity and need surgery for replacement, which is prone to cause infection and bring a lot of inconvenience to the lives of patients. Wireless power transfer can eliminate the dependence on percutaneous wires, and the service life of implanted devices can avoid becoming limited by battery capacity, thus producing a very potential power supply method. Over the past few decades, several wireless power transfer techniques have been proposed for powering implanted medical devices, including inductive coupling [2], capacitive coupling [3,4], ultrasound [5,6] and far-field electromagnetic radiation [7]. In general, inductive coupling and capacitive coupling techniques consume less volume, while inductive coupling and ultrasonic transmission are more efficient. In addition, inductive coupling has less tissue absorption and is more liable to transfer higher power [8]. Among these techniques, inductive coupling has been widely studied in the field of wireless power transfer for implantable medical devices due to its effectiveness, robustness and safety.

In neural recording applications, the implanted coil needs to be mounted between the scalp and skull of the human head, so planar spiral coils become the preferred coil form due to the limitation of implantable space [9,10]. The electrode array used to capture neuronal activity in the brain region contains at least hundreds of electrodes, and the captured data need to be transmitted wirelessly to the outside of the body in real time, resulting in the overall power consumption of the implanted device generally being around 10 mW. In addition, wireless power transfer must meet the specific absorption rate (SAR) requirements for biological safety, otherwise it may cause the temperature of local tissues of the human body to rise, bring discomfort to patients or cause other complications [11]. For example, the United States stipulates that the average radio frequency (RF) power absorbed per 1 g of human tissue shall not exceed 1.6 W/kg, while the European standard stipulates a SAR limit of 2 W/kg per 10 g of human tissue on average [12]. Considering the size limitations, load power requirements and biological safety requirements of human tissues for implantable devices, the transmitted power of the primary coil outside of the body cannot be arbitrarily increased. Instead, the system power transfer efficiency can only be improved as much as possible to meet the power transfer requirements.

**Figure 1:**
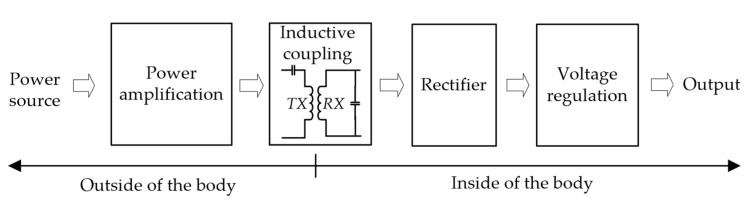
Block diagram of inductive coupling wireless power transfer system.

As shown in Figure 1, the wireless power transfer (WPT) system of the implantable neural recording microsystem mainly consists of a power amplification module located outside of the body, a rectifier module and a voltage regulation module implanted in the body, and an inductive coupling module that transfers energy from outside of the body to the inside. However, the power transfer efficiency of the inductive coupling module is usually the bottleneck that restricts the transfer efficiency of the whole system.

Various techniques have been proposed to improve system efficiency. One such technique is the optimization of coil geometric parameters, which has been described to increase the quality factor and coupling coefficient of the coupling coils. This in turn improves the power transfer efficiency for a given load [13,14]. However, the entire coil optimization process has to be rerun once the load changes. Three-coil and four-coil structures have been proposed to improve system efficiency, but additional coils may lead to oversized implants, especially when implantable space is very limited [15,16]. It has been found that an optimal load impedance exists that maximizes the system transfer efficiency, and an actual load can be transformed into this optimal load via impedance transformation [17,18,19]. In addition to coil optimization, there are some other techniques, such as adding a nanocrystalline core to reduce eddy current loss [20], using switch-controlled capacitors to realize variable capacitance for self-tuning [21] and utilizing joint control with variable zero-voltage switching angle for dynamic optimization [22]. However, those techniques are not preferred for neural recording applications as they may lead to potential biocompatibility or excessive volume issues, especially given the harsh height constraints.

This paper presents a design and optimization method for planar spiral inductors which can meet the demanding requirements of neural recording applications. Unlike previous work, this method aims to maximize the power transfer efficiency of inductive coupling, and more importantly, the entire optimization process is not limited to a specific given load. The modeling of inductive coupling is first introduced, which simplifies system modeling by combining semi-empirical formulas with theoretical models, thus saving a lot of effort and resources. Then, the design and optimization process of planar spiral coils is analyzed in detail. The power transfer efficiency is decoupled from the specific load impedance through the optimal resonant load transformation, so that the actual load change no longer requires rerunning the coil optimization process, only adjustments to the impedance transformation network. Finally, the modeling calculation, electromagnetic simulation and measurement results are compared.

## 2. Modeling of Inductive Coupling

**Figure 2:**
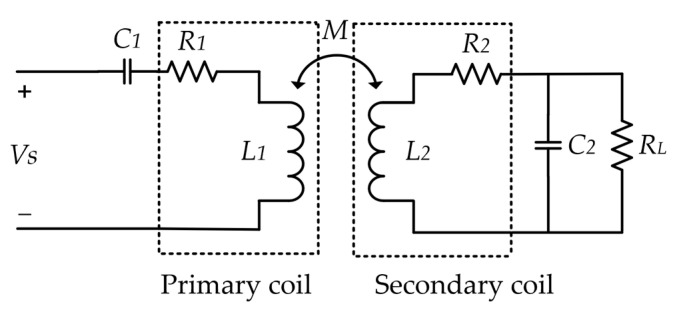
Inductive coupling equivalent circuit model.

A general equivalent circuit model of inductive coupling is shown in Figure 2. $L_{1}$, $R_{1}$, $L_{2}$ and $R_{2}$ are the self-inductance and parasitic resistance of the primary and secondary coils, respectively, and M is the mutual inductance between the coils. The introduction of $C_{1}$ and $C_{2}$ makes the primary coil and the secondary coil work at the same resonant angle frequency $\omega_{0}$, respectively, in series resonance and parallel resonance states, improving the power transfer efficiency. $R_{L}$ represents the equivalent load, and $V_{s}$ represents the driving source.

The power transfer efficiency η of inductive coupling is expressed as [15]:

(1) $$\eta =\frac{k^{2}Q_{1}Q_{2L}}{1+k^{2}Q_{1}Q_{2L}}\frac{Q_{2L}}{Q_{L}}$$

where $Q_{1}=\frac{\omega_{0}L_{1}}{R_{1}}$, $Q_{2}=\frac{\omega_{0}L_{2}}{R_{2}}$, are the quality factors of the primary and secondary coils, respectively; $Q_{L}=\frac{R_{L}}{\omega_{0}L_{2}}$ is the loaded quality factor of the secondary coil and $Q_{2L}=\frac{Q_{2}Q_{L}}{Q_{2}+Q_{L}}$. $k=\frac{M}{\sqrt{L_{1}L_{2}}}$ is the coupling coefficient between the coils, which is related to the mutual inductance and self-inductance of the coils.

### 2.1. Maximum Power Transfer Efficiency and Optimal Load Condition

Based on the general inductive coupling structure, when the load meets the optimal load condition:

(2) $$Z_{L}^{opt}=R_{L,opt}+jX_{L,opt}=\frac{\omega_{0}L_{2}\sqrt{1+k^{2}Q_{1}Q_{2}}}{Q_{2}}-j\omega_{0}L_{2}$$

Theoretically, the maximum power transfer efficiency $\eta_{max}$ that can be obtained by inductive coupling is [23]

(3) $$\eta_{max}=\frac{k^{2}Q_{1}Q_{2}}{{\left(1+\sqrt{1+k^{2}Q_{1}Q_{2}}\right)}^{2}}$$

It can be seen from Equation (3) that this theoretical limit depends on k and Q of the coupling coils and is independent of the actual load. To improve the power transfer efficiency of inductive coupling, the quality factors and coupling coefficient of the coils should be maximized as much as possible.

### 2.2. Planar Spiral Coil Modeling

The following coil modeling is simplified by combining semi-empirical formulas with theoretical models. This can save significant resources and effort required in the subsequent optimization process.

**Figure 3:**
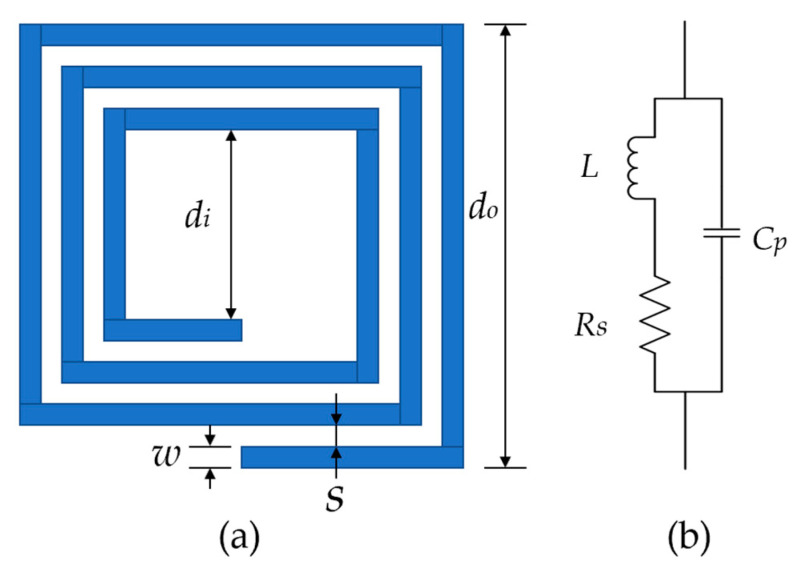
(**a**) Schematic diagram of a square planar spiral coil; (**b**) coil equivalent model.

A square planar spiral coil on a printed circuit board (PCB) is shown in Figure 3a. Its main geometric parameters include conductor line width w, line space s, number of turns N, outer diameter $d_{o}$, inner diameter $d_{i}$ and conductor thickness $t_{c}$. The equivalent model is shown in Figure 3b. The quality factor Q of the coil can be derived as:

(4) $$Q=\frac{\omega L-\omega \left(R_{s}^{2}+\omega^{2}L^{2}\right)C_{P}}{R_{s}}$$

where ω is the operating angular frequency of the coil, L is the self-inductance, $C_{P}$ is the parasitic capacitance and $R_{S}$ is the parasitic resistance.

The self-inductance of the planar spiral coil can be derived from the semi-empirical formula [24,25]:

(5) $$L=\frac{1.27\cdot \mu_{0}\cdot N^{2}\cdot d_{avg}}{2}\left[\ln\left(\frac{2.07}{\varphi }\right)+0.18\varphi +0.13\varphi^{2}\right]$$

where $\mu_{0}$ is the permeability of space, and φ is the coil fill factor, $\varphi =\frac{d_{o}-d_{i}}{d_{o}+d_{i}},d_{avg}=(d_{o}-d_{i})/2$.

The parasitic capacitance of the planar spiral coil mainly includes two parts, the capacitance between the adjacent conductors $C_{pc}$ and the capacitance between the conductor and the substrate $C_{ps}$. For a typical FR4 substrate, the parasitic capacitance can be calculated using the following semi-empirical formula [13]:

(6) $$C_{P}=C_{pc}+C_{ps}\approx \left(0.9+0.1\varepsilon_{rs}\right)\varepsilon_{0}\frac{t_{c}}{s}l_{g}$$

where $\varepsilon_{rs}$ is the relative dielectric constant of the substrate, $\varepsilon_{0}$ is the vacuum dielectric constant, $t_{c}$ is the conductor thickness and $l_{g}$ is the total length of the gap between the coil conductors, $l_{g}=4\left(d_{o}-wN\right)\left(N-1\right)-4sN\left(N-1\right)$.

The equivalent resistance $R_{S}$ of the planar spiral coil can be given using the following equation [26]:

(7) $$R_{S}=R_{DC}\xi \left(\Delta_{1}+\frac{3\cdot Nw}{\left[\left(Nw\right)+\left(N-1\right)s\right]\cdot \Delta_{2}}\right)$$

where $R_{DC}$ is the direct current (DC) resistance of the conductor; $R_{DC}=\frac{\rho l_{c}}{wt_{c}}$, ρ is the resistivity of the conductor; $l_{c}$ is the total length of the coil conductor, with $l_{c}=4Nd_{o}-4Nw-{\left(2N+1\right)}^{2}\left(s+w\right)$; $\xi =\sqrt{\frac{Nw}{\left(Nw\right)+\left(N-1\right)s}}\cdot \frac{t_{c}}{\delta }$; δ is the the skin depth of the conductor; $\delta =\sqrt{\frac{2\rho }{\omega \cdot \mu }}$ and μ is the conductor permeability; $\Delta_{1}$ and $\Delta_{2}$ characterize the skin effect and proximity effect of the conductor, respectively [27], with $\Delta_{1}=\frac{\sinh\left(2\xi \right)+\sin\left(2\xi \right)}{\cosh\left(2\xi \right)-\cos\left(2\xi \right)}$, $\Delta_{2}=2\frac{\sinh\left(2\xi \right)-\sin\left(2\xi \right)}{\cosh\left(2\xi \right)+\cos\left(2\xi \right)}$.

The mutual inductance M between the inductively coupled primary and secondary coils can be calculated using the following equations [28]:

(8) $$M=1.1\sum_{i=1}^{N}\sum_{j=1}^{N}M_{ij}$$

(9) $$M_{ij}=\frac{2\mu_{m}}{\alpha }\sqrt{r_{i}r_{j}}\left[\left(1-\frac{\alpha^{2}}{2}\right)K\left(\alpha \right)-E\left(\alpha \right)\right]$$

(10) $$\alpha =2\sqrt{\frac{r_{i}r_{j}}{{\left(r_{i}+r_{j}\right)}^{2}+D^{2}}}$$

where $M_{ij}$ represents the mutual inductance between the i-th turn of the primary coil and the j-th turn of the secondary coil, and the total mutual inductance M can be obtained by adding the mutual inductance values between each turn on the primary coil and all turns of the secondary coil. $\mu_{m}$ is the permeability of the medium, $K\left(\alpha \right)$ and E(α) are complete elliptic integrals of the first and second kind, respectively [29,30], $r_{i}$ and $r_{j}$ are the radii of the i-th turn of the primary coil and the j-th turn of the secondary coil, respectively, and D is the distance between these two turns.

## 3. Design and Optimization of Planar Spiral Coils

### 3.1. Design Constraints

Coil design should take into account both application requirements and manufacturing process constraints. In neural recording applications, the size of the implanted coil should be as small as possible to reduce discomfort and potential risk to the recipient. Especially considering that the implanted coil is mounted subcutaneously with extremely limited headroom between the scalp and skull, the outer diameter of the secondary coil is set to 10 mm, and a 0.8-mm thick FR4 substrate and 1 oz copper are chosen. The minimum values of the line width w and spacing s of the coil donductor are both set to 0.15 mm due to PCB manufacturing constraints. In addition, the working distance between the external and implanted coils is 10 mm, and the operating frequency is selected as 13.56 MHz in the ISM band according to system design requirements. The above design constraints are summarized in Table 1.

### 3.2. Optimization Process

The optimization of coil geometry involves a tradeoff among multiple variables that require repeated iterations. For example, maintaining a minimum line space, increasing the number of turns of the coil and reducing the line width can increase the inductance value of the coil with the same outer diameter, but the quality factor of the coil may decrease due to the increase in coil resistance.

**Figure 4:**
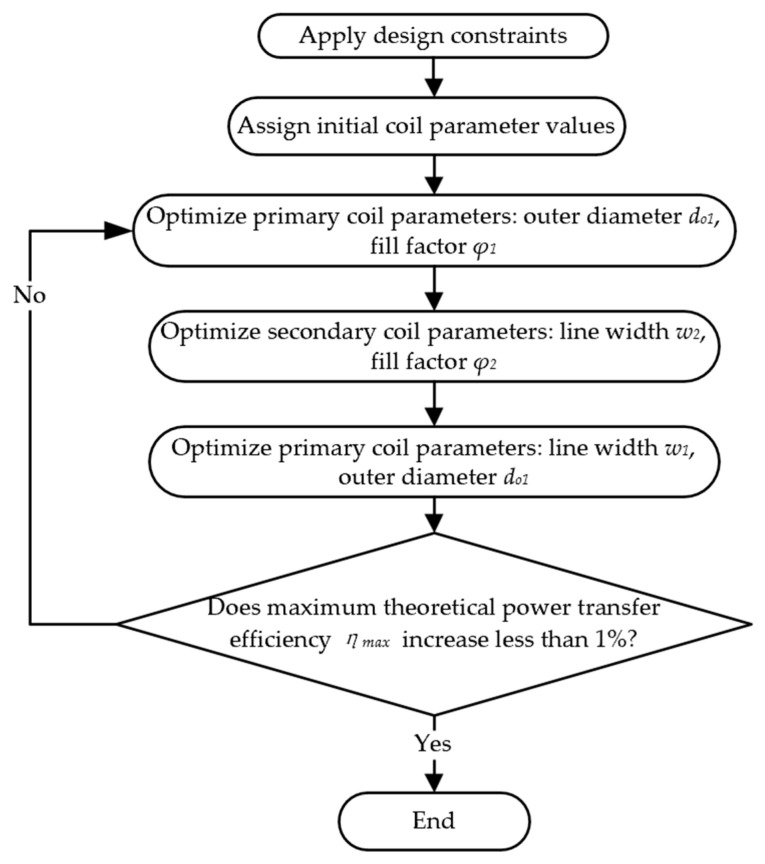
Flowchart of the planar spiral coil optimization.

The literature [13] gives a complete coil design flow for a given load, based on (1) deriving the maximum power transfer efficiency for that load. However, once the load changes, the entire process needs to be rerun. In contrast, this paper decouples the coil optimization from an actual load by introducing the optimal resonant load transformation to convert the actual load to the optimal load that satisfies. (2) As a result, the coil optimization process is no longer specific to a particular load. When the actual load changes, the optimization process does not need to be rerun entirely. Instead, only the impedance transformation network needs to be updated. Moreover, this approach uses (3) the objective function and has the advantage of a clear optimization goal. Whether the algorithm iteration is reasonable can be easily judged by whether it is approaching the theoretical limit. This is different from the previous work, which only aims to improve k and Q as much as possible, and the final result depends on the effectiveness of the iterative algorithm. Figure 4 shows a flowchart using (4)–(10) to design and optimize the coils.

#### 3.2.1. Optimize the Outer Diameter $d_{o1}$ and Fill Factor $\varphi_{1}$ of the Primary Coil

**Figure 5:**
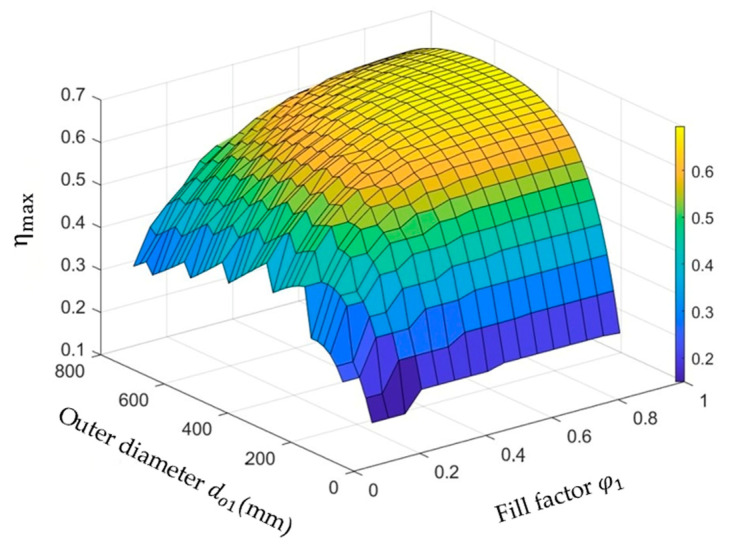
Optimize the outer diameter $d_{o1}$ and fill factor $\varphi_{1}$ of the primary coil.

Sweep $d_{o1}$ and $\varphi_{1}$ by keeping $w_{1}$ and $s_{1}$ unchanged, and the relationship between the maximum power transfer efficiency $\eta_{max}$ and the variables $d_{o1}$ and $\varphi_{1}$ is shown in Figure 5.

Within a certain range, the $\eta_{max}$ increases with the increase in $d_{o1}$ and $\varphi_{1}$. When $\varphi_{1}$ > 0.4, the efficiency rise flattens out, which means that the number of turns near the center of the coil does not help much to improve efficiency. When $d_{o1}$ = 40 mm and $\varphi_{1}$ = 0.465, $\eta_{max}$ = 66.9%.

#### 3.2.2. Optimize the Line Width $w_{2}$ and Fill Factor $\varphi_{2}$ of the Secondary Coil

**Figure 6:**
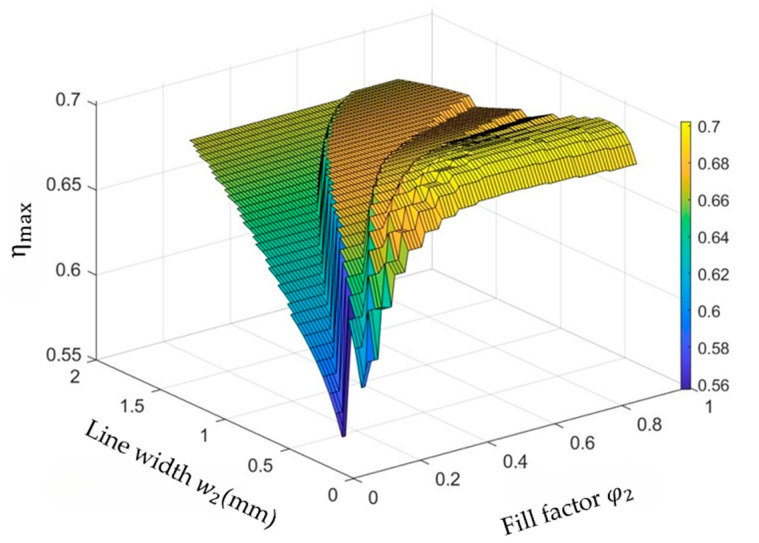
Optimize the line width $w_{2}$ and fill factor $\varphi_{2}$ of the secondary coil.

By fixing $d_{o2}$ = 10 mm and sweeping $w_{2}$ and $\varphi_{2}$ of the secondary coil, the trend of $\eta_{max}$ can be observed in Figure 6. The maximum value of $\eta_{max}$ is obtained when $w_{2}$ = 0.3 mm and $\varphi_{2}$ = 0.3, which is about 70.24%. Similarly, when $\varphi_{2}$ > 0.3, the efficiency does not change much.

#### 3.2.3. Optimize the Outer Diameter $d_{o1}$ and Line Width $w_{1}$ of the Primary Coil

**Figure 7:**
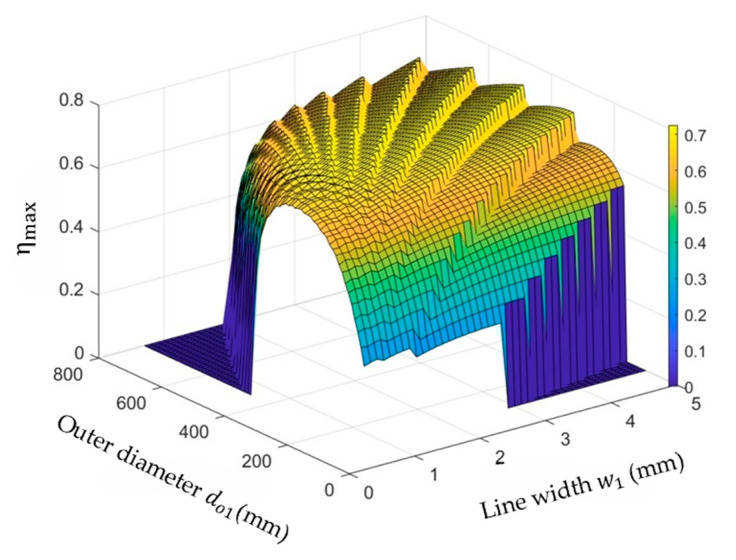
Optimize the outer diameter $d_{o1}$ and line width $w_{1}$ of the primary coil.

After optimizing the secondary coil, the line width and outer diameter of the primary coil can be further tuned to boost the maximum power transfer efficiency, since the size of the primary coil is less constrained. Increasing $w_{1}$ can reduce $R_{s1}$ and increase $Q_{1}$, and also result in larger $d_{o1}$ while keeping N constant. The change in $\eta_{max}$ with $d_{o1}$ and $w_{1}$ is shown in Figure 7. When $d_{o1}$ = 40 mm and $w_{1}$ = 1.3 mm, $\eta_{max}$ = 72.2%.

Each step of the optimization process requires iterations until the improvement in $\eta_{max}$ of each iteration is less than 1%. Table 2 summarizes the optimized coil geometry parameters obtained according to the optimization process described above.

## 4. Optimal Resonant Load Transformation

The secondary coil loop in a parallel resonance state can be equivalent to a current source with internal resistance. When the load impedance $R_{L}$ is small, it is beneficial for the load to extract more energy from the secondary coil, but it will reduce the loaded quality factor of the secondary coil, which is not conducive to the coil to maintain resonance; conversely, when $R_{L}$ is large, the secondary coil has a higher loaded quality factor, but it is not conducive to the load-extracting energy from the current source. Inductive coupling achieves the maximum power transfer efficiency only when $R_{L}$ satisfies the optimal load condition in (2). In the traditional resonant coupling structure, the resonant capacitor can only achieve the imaginary part of the desired optimal load, so η cannot reach $\eta_{max}$, and it is also heavily dependent on $R_{L}$. Once the load changes, the transfer efficiency may be greatly reduced in practical applications [23].

In order to make the equivalent load seen by the secondary coil loop close to the optimal load, an impedance transformation network is inserted between the secondary coil and the actual load to achieve the optimal resonant load transformation. In this way the actual load impedance is converted to the optimal load given in (2), which not only keeps the secondary coil resonant with a high Q value, but also facilitates the load to extract energy from the equivalent current source [19]. This is different from traditional conjugate impedance matching. Conjugate matching aims to reduce energy reflection on the transmission line, and the theoretical upper limit of transmission efficiency is only 50% [17]. In addition, the three-coil or four-coil structures proposed in the literature [15,16] essentially implement load transformation by introducing additional coils to reduce the influence of load and source impedance on the power transfer efficiency, but it will obviously increase the device size, which is severely limited by implantable space and difficult to adjust.

**Figure 8:**
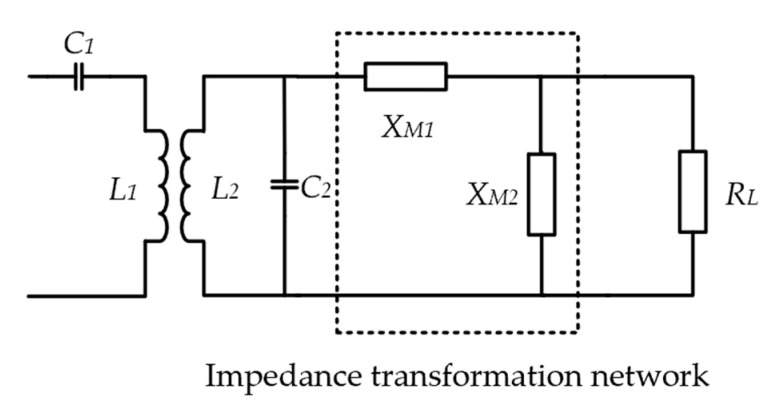
L-shaped network for optimal resonant load transformation.

As shown in Figure 8, an L-type impedance transformation network is inserted into the secondary loop to achieve the optimal resonant load transformation. By combining with (2), (11) and (12) can be easily derived, which can be used to calculate the values of the two components, $X_{M1}$ and $X_{M2}$.

(11) $$R_{L,opt}=\frac{\omega L\sqrt{1+k^{2}Q_{1}Q_{2}}}{Q_{2}}=Re\left(jX_{M1}+\frac{jX_{M2}\cdot R_{L}}{jX_{M2}+R_{L}}\right)$$

(12) $$X_{L,opt}=-\omega L=Im\left(jX_{M1}+\frac{jX_{M2}\cdot R_{L}}{jX_{M2}+R_{L}}\right)$$

**Figure 9:**
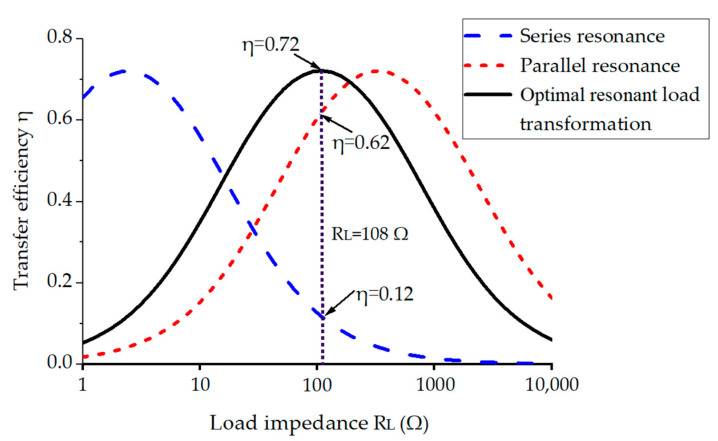
Simulation comparison of the optimal resonant load transformation with conventional series and parallel resonance.

Assuming that the neural recording microsystem requires a maximum power of 30 mW and a regulated voltage of 1.8 V, the equivalent load impedance is 108 Ω. Based on the parameters in Table 2, $X_{M1}$ and $X_{M2}$ can be calculated as a series capacitance of 962.04 pF and a parallel capacitance of 720.88 pF, respectively [31]. Figure 9 compares the simulated efficiency based on the optimal load transformation with conventional series and parallel resonant coupling. Obviously, due to the loading effect, series resonant coupling is only more efficient when $R_{L}$ is small, while parallel resonant coupling is only more efficient when $R_{L}$ is large. The presented scheme can achieve much higher efficiency for this particular given load.

## 5. Electromagnetic Simulation and Test Verification

The coils with the geometric parameters in Table 2 have been modeled using the commercial three-dimensional electromagnetic simulation software HFSS and fabricated for testing. The electrical parameters of the coils can be obtained through simulation, from which k and η of the inductive coupling can be calculated.

**Figure 10:**
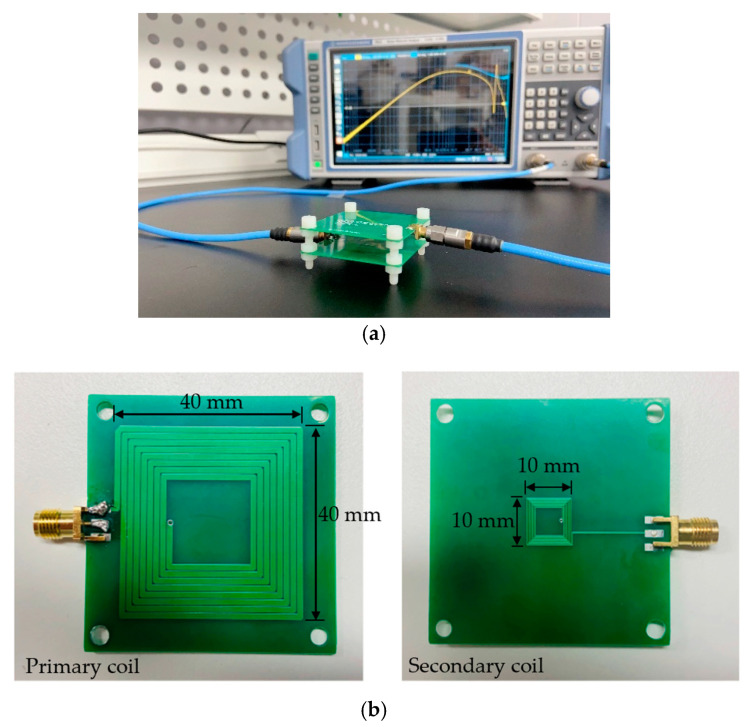
(**a**) Experimental setup of inductive coupling measurement; (**b**) fabricated primary and secondary coils.

The fabricated PCB coils and the inductive coupling test setup are shown in Figure 10. Two-port S-parameters of the inductive coupling are first measured using a vector network analyzer (VNA), and then converted to Z-parameters. This allows the electrical parameters of the coils to be easily extracted according to the following equations:

(13) $$L_{i}=\frac{Im\left(Z_{ii}\right)}{2\pi f},\;\;\;\;\;\;i=1,2$$

(14) $$R_{i}=Re(Z_{ii}),\;\;\;\;\;\;i=1,2$$

(15) $$Q_{i}=\frac{Im\left(Z_{ii}\right)}{Re\left(Z_{ii}\right)},\;\;\;\;\;\;i=1,2$$

(16) $$M=\frac{Im\left(Z_{21}\right)}{2\pi f}$$

**Figure 11:**
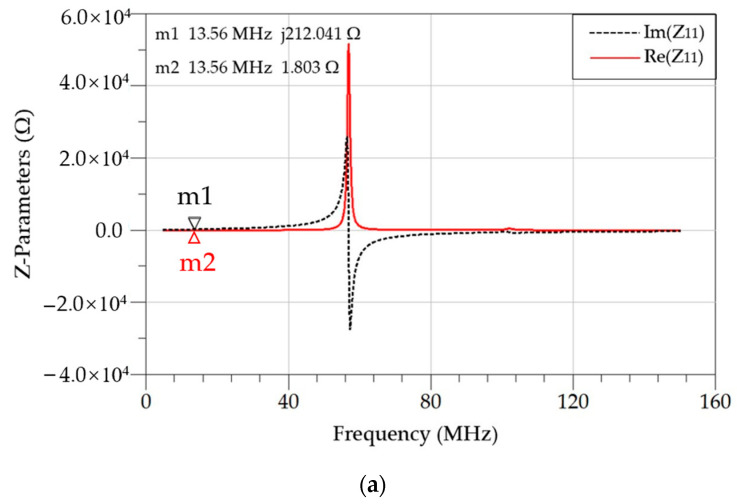

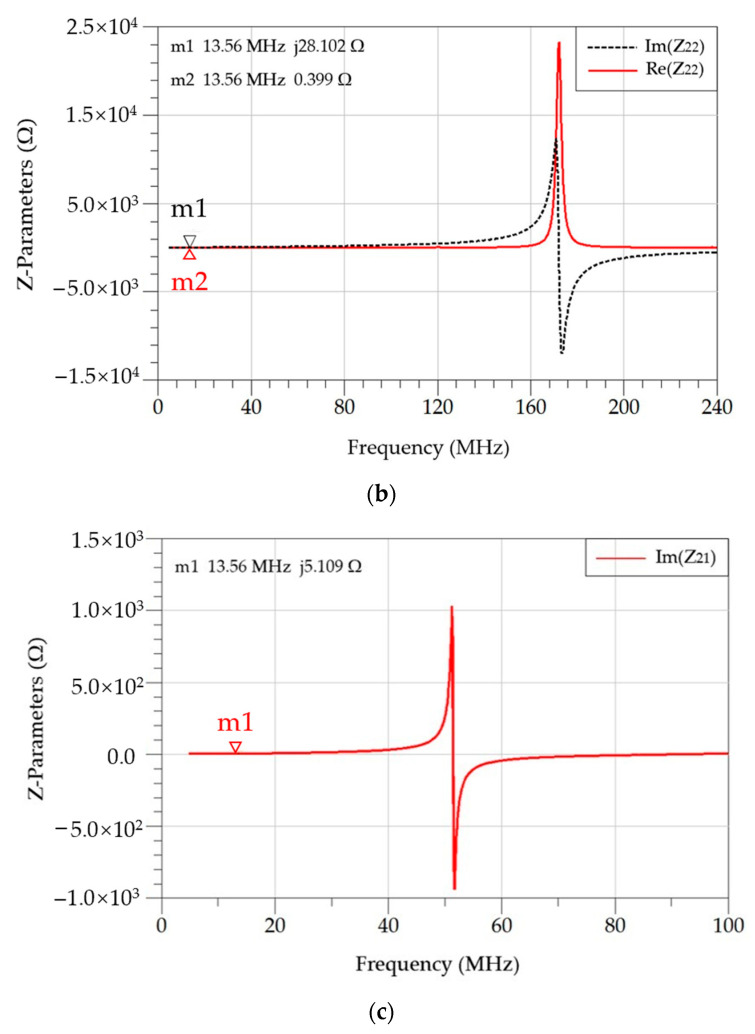
Measured Z-parameters. (**a**) The real and imaginary part curves of $Z_{11}$ of the primary coil; (**b**) the real and imaginary part curves of $Z_{22}$ of the secondary coil; (**c**) the imaginary part curve of $Z_{21}$, representing the mutual inductance.

The measured Z-parameters are shown in Figure 11, and the extracted electrical parameters are listed in Table 3. It is worth noting that the frequency point where the sharp peak of the real part curve and the abrupt transition from positive to negative of the imaginary part curve of the Z-parameters indicates the self-resonant frequency (SRF) of the coil. It can be seen that the operating frequency of 13.56 MHz is at least three times lower than the SRF for both the primary and secondary coils, which means the coils can work stably at the operating frequency.

Table 3 compares the modeling calculations, three-dimensional electromagnetic simulation and measurement results. Compared with the measured results, the electrical parameters obtained from the modeling calculations, including the self-inductance and quality factor of the primary and secondary coils, as well as the coupling coefficient between the two coils, have a maximum difference of less than 11%. There is also a slight deviation between the parameters obtained via simulation and the measured values due to some trade-offs between accuracy and speed in the process of three-dimensional electromagnetic simulation. Further substituting these parameters into Equation (3), the theoretical maximum power transfer efficiency based on the modeling parameters is 72.2%, which is pretty close to 71.9% based on the measured parameters. This confirms the effectiveness of the presented optimization method, which focuses on the maximum theoretical power transfer efficiency and decouples it from actual loads. At the same time, combining semi-empirical formulas with theoretical models greatly saves the resources and efforts required for the optimization process.

To directly measure the efficiency and further verify the effectiveness of the optimal resonant load transformation, the two ports of the VNA are connected to the primary and secondary coils of the inductive coupling system, respectively. In this way, the source impedance and load impedance of the system will be the port impedance of the VNA, which is 50 ohms, and the power transfer efficiency can be directly obtained by measuring the $S_{21}$ parameter. Then, an impedance-matching network is added to the primary coil side to reduce input energy reflection, and an L-type network is inserted between the secondary coil and the VNA input to convert the 50-ohm load into the optimal resonant load. In this way, the measured power transfer efficiency should be close to the theoretical limit mentioned above. This measurement method is more stable and repeatable than probing the voltage at 13.56 MHz with an oscilloscope, a method described in the literature.

**Figure 12:**
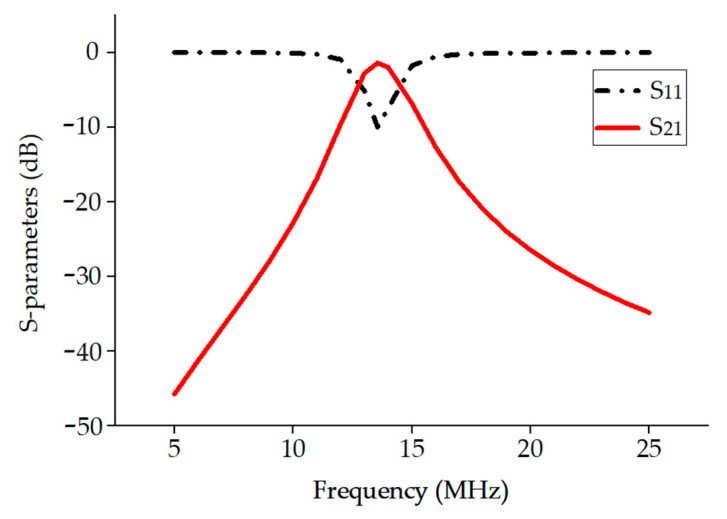
$S_{11}$ and $S_{21}$ parameters at a coupling distance of 10 mm.

For the fabricated inductive coupling coils mentioned above, the optimal load impedance can be calculated as 2.4–j28.12 Ω from (2). Substituting it into (11) and (12) yields the required L-type network consisting of a series capacitor $X_{M1}$ = 673.32 pF and a parallel capacitor $X_{M2}$ = 1.05 nF. These two capacitors are soldered onto the feed path on the back of the PCB to avoid their impact on the inductive coupling. As shown in Figure 12, when the coupling distance is 10 mm, the measured $S_{21}$ is −1.55 dB, corresponding to a power transfer efficiency of 70%. This efficiency is very close to the above theoretical limit of 71.9%, which validates the effectiveness of the optimal resonant load transformation.

## 6. Conclusions

This paper discusses the design and optimization method of inductively coupled coils for implantable neural recording microsystems. Considering that the coil size is strictly limited by the implantable space, the high-power demand of the implantable system and the safety requirements of biological tissues, the power transfer efficiency of inductive coupling is often the bottleneck of the entire wireless power transfer system. Thus, this paper presents the coil design and optimization process in detail with the aim of maximizing the power transfer efficiency. The semi-empirical formulas are combined with the theoretical models to simplify the modeling of inductive coupling, and the coil optimization is decoupled from a specific load through the optimal resonant load transformation. The optimization uses the maximum theoretical power transfer efficiency as the objective function. Once the actual load changes, there is no need to rerun the entire optimization process. Instead, only updating the load transformation network is necessary. Square planar spiral coils have been designed and fabricated given the challenges of powering the neural recording microsystem. The modeling calculation, electromagnetic simulation and measurement results have been compared. The measured power transfer efficiency of inductive coupling is 70%, which is close to the theoretical limit of 71.9%. This confirms the effectiveness of the proposed method.

## Figures and Tables

**Table 1:** Design constraints.

Parameters	Symbols	Values
Minimum line width	w	0.15 mm
Minimum line space	s	0.15 mm
Conductor thickness	$t_{c}$	1 oz
Secondary coil outer diameter	$d_{o2}$	10 mm
FR4 substrate thickness	$t_{s}$	0.8 mm
Working distance	D	10 mm
Operating frequency	f	13.56 MHz

**Table 2:** Optimized coil geometry parameters.

Parameters	Primary Coil	Secondary Coil
Outer diameter $d_{o}$ (mm)	40	10
Inner diameter $d_{i}$ (mm)	14.60	5.55
Turns N	8	5
Line space s (mm)	0.15	0.15
Line width w (mm)	1.3	0.3

**Table 3:** Comparison of the modeling calculation, electromagnetic simulation and measured results.

Parameters	Modeling Calculation	Electromagnetic Simulation	Measured Results
$L_{1}$(μH)	2.22	2.27	2.49
$R_{s1}(\Omega )$	1.53	1.69	1.80
$Q_{1}$	119	114	118
$L_{2}$(μH)	0.310	0.320	0.330
$R_{s2}(\Omega )$	0.470	0.420	0.399
$Q_{2}$	60.0	64.0	70.4
M(nH)	59.6	59.8	60.0
k	0.0721	0.0699	0.0662
$\eta_{max}$ by Equation (3)	72.2%	71.8%	71.9%

## Data Availability

Not applicable.

## Abbreviations

A summary of the symbols used in the article:

**Symbols**	**Description**
$d_{o}$	Coil outer diameter
$d_{i}$	Coil inner diameter
w	Conductor line width
s	Conductor line space
N	Number of turns
φ	Coil fill factor
L	Self-inductance
$C_{P}$	Equivalent parasitic capacitance
$R_{S}$	Equivalent parasitic resistance
k	Coupling coefficient
Q	Quality factor
η	Power transfer efficiency
$\eta_{max}$	Maximum theoretical power transfer efficiency
$Z_{L}^{opt}$	Optimal load
$V_{s}$	Driving voltage source
$R_{L}$	Equivalent load
$\omega_{0}$	Resonant angle frequency
$\mu_{0}$	Vacuum permeability
$t_{c}$	Conductor thickness
$\varepsilon_{rs}$	Relative dielectric constant of substrate
$\varepsilon_{0}$	Vacuum permittivity
$l_{g}$	Total length of gap between coil conductors
$R_{DC}$	Direct current resistance of conductor
ρ	Conductor resistivity
$l_{c}$	Conductor length
δ	Skin depth of conductor
μ	Conductor permeability
$\Delta_{1}$	Characterizing conductor skin effect
$\Delta_{2}$	Characterizing conductor proximity effect
M	Mutual inductance
$M_{ij}$	Mutual inductance between the i-th turn of primary coil and the j-th turn of secondary coil
$\mu_{m}$	Medium permeability
$K\left(\alpha \right)$	Complete elliptic integral of the first kind
E(α)	Complete elliptic integral of the second kind
$r_{i}$	Radius of the i-th turn of primary coil
$r_{j}$	Radius of the j-th turn of secondary coil
$t_{s}$	FR4 substrate thickness
D	Working distance
f	Operating frequency

## References

[1] Mahfouz M.R., Bashirullah R. (2010). 2011 IEEE Topical Meeting on Biomedical Radio and Wireless Technologies, Networks, and Sensing Systems. IEEE Microw. Mag..

[2] Lee B., Ghovanloo M. (2019). An Overview of Data Telemetry in Inductively Powered Implantable Biomedical Devices. IEEE Commun. Mag..

[3] Jegadeesan R., Agarwal K., Guo Y.X., Yen S.C., Thakor N.V. (2017). Wireless Power Delivery to Flexible Subcutaneous Implants Using Capacitive Coupling. IEEE Trans. Microw. Theory Tech..

[4] Sodagar A.M., Amiri P. Capacitive coupling for power and data telemetry to implantable biomedical microsystems. Proceedings of the 2009 4th International IEEE/EMBS Conference on Neural Engineering.

[5] Hong Y., Jin L., Wang B., Liao J., He B., Yang T., Long Z., Li P., Zhang Z., Liu S. (2021). A wood-templated unidirectional piezoceramic composite for transmuscular ultrasonic wireless power transfer. Energy Environ. Sci..

[6] Jiang L., Yang Y., Chen Y., Zhou Q. (2020). Ultrasound-Induced Wireless Energy Harvesting: From Materials Strategies to Functional Applications. Nano Energy.

[7] Liu C., Guo Y.X., Xiao S. (2012). A Hybrid Patch/Slot Implantable Antenna for Biotelemetry Devices. IEEE Antennas Wirel. Propag. Lett..

[8] Khan S.R., Pavuluri S.K., Cummins G., Desmulliez M.P.Y. (2020). Wireless Power Transfer Techniques for Implantable Medical Devices: A Review. Sensors.

[9] Liu X., Zhang M., Xiong T., Richardson A.G., Lucas T.H., Chin P.S., Etienne-Cummings R., Tran T.D., Van der Spiegel J. (2016). A Fully Integrated Wireless Compressed Sensing Neural Signal Acquisition System for Chronic Recording and Brain Machine Interface. IEEE Trans. Biomed. Circuits Syst..

[10] Seung Bae L., Hyung-Min L., Kiani M., Uei-Ming J., Ghovanloo M. (2010). An Inductively Powered Scalable 32-Channel Wireless Neural Recording System-on-a-Chip for Neuroscience Applications. IEEE Trans. Biomed. Circuits Syst..

[11] (2019). IEEE Approved Draft Standard for Safety Levels with Respect to Human Exposure to Electric, Magnetic and Electromagnetic Fields, 0 Hz to 300 GHz.

[12] Pozar D.M. (2011). Microwave Engineering.

[13] Uei-Ming J., Ghovanloo M. (2007). Design and optimization of printed spiral coils for efficient transcutaneous inductive power transmission. IEEE Trans. Biomed. Circuits Syst..

[14] Uei-Ming J., Ghovanloo M. (2009). Modeling and optimization of printed spiral coils in air, saline, and muscle tissue environments. IEEE Trans. Biomed. Circuits Syst..

[15] Kiani M., Jow U.M., Ghovanloo M. (2011). Design and Optimization of a 3-Coil Inductive Link for Efficient Wireless Power Transmission. IEEE Trans. Biomed. Circuits Syst..

[16] Ramrakhyani A.K., Mirabbasi S., Mu C. (2011). Design and optimization of resonance-based efficient wireless power delivery systems for biomedical implants. IEEE Trans. Biomed. Circuits Syst..

[17] Zargham M., Gulak P.G. (2012). Maximum achievable efficiency in near-field coupled power-transfer systems. IEEE Trans. Biomed. Circuits Syst..

[18] Xue R.-F., Cheng K.-W., Je M. (2013). High-Efficiency Wireless Power Transfer for Biomedical Implants by Optimal Resonant Load Transformation. IEEE Trans. Circuits Syst. I Regul. Pap..

[19] Miao Z., Liu D., Gong C. (2017). Efficiency Enhancement for an Inductive Wireless Power Transfer System by Optimizing the Impedance Matching Networks. IEEE Trans. Biomed. Circuits Syst..

[20] Yang F., Jiang J., Sun C., He A., Chen W., Lan Y., Song K. (2022). Efficiency Improvement of Magnetic Coupler with Nanocrystalline Alloy Film for UAV Wireless Charging System with a Carbon Fiber Fuselage. Energies.

[21] Zhang J., Zhao J., Zhang Y., Deng F. (2020). A Wireless Power Transfer System with Dual Switch-Controlled Capacitors for Efficiency Optimization. IEEE Trans. Power Electron..

[22] Jiang Y., Wang L., Fang J., Zhao C., Wang K., Wang Y. (2020). A Joint Control with Variable ZVS Angles for Dynamic Efficiency Optimization in Wireless Power Transfer System. IEEE Trans. Power Electron..

[23] Ko W.H., Liang S.P., Fung C.D.F. (1977). Design of radio-frequency powered coils for implant instruments. Med. Biol. Eng. Comput..

[24] Mohan S.S., del Mar Hershenson M., Boyd S.P., Lee T.H. (1999). Simple accurate expressions for planar spiral inductances. IEEE J. Solid-State Circuits.

[25] Zolog M., Pitica D., Pop O. Characterization of Spiral Planar Inductors Built on Printed Circuit Boards. Proceedings of the 2007 30th International Spring Seminar on Electronics Technology (ISSE).

[26] Khan S.R., Pavuluri S.K., Desmulliez M.P. New analytical model for the characterisation of printed spiral coils for wireless power transfer. Proceedings of the 12th European Conference on Antennas and Propagation (EuCAP 2018).

[27] Ferreira J.A. (1994). Improved analytical modeling of conductive losses in magnetic components. IEEE Trans. Power Electron..

[28] Zierhofer C.M., Hochmair E.S. (1996). Geometric approach for coupling enhancement of magnetically coupled coils. IEEE Trans. Biomed. Eng..

[29] Soma M., Galbraith D.C., White R.L. (1987). Radio-Frequency Coils in Implantable Devices: Misalignment Analysis and Design Procedure. IEEE Trans. Biomed. Eng..

[30] Atluri S., Ghovanloo M. Design of a Wideband Power-Efficient Inductive Wireless Link for Implantable Biomedical Devices Using Multiple Carriers. Proceedings of the Conference Proceedings 2nd International IEEE EMBS Conference on Neural Engineering.

[31] Stoecklin S., Yousaf A., Volk T., Reindl L. (2016). Efficient Wireless Powering of Biomedical Sensor Systems for Multichannel Brain Implants. IEEE Trans. Instrum. Meas..

